# Interrupted social activities and burnout in caregivers: The role of activity value

**DOI:** 10.1093/geroni/igaf122.1895

**Published:** 2025-12-31

**Authors:** Jeong Eun Lee, Eunbea Kim, Yulri Kim, Joseph Svec

**Affiliations:** Iowa State University, Ames, Iowa, United States; Auburn University, Auburn, Alabama, United States; Iowa State University, Ames, Iowa, United States; St. Joseph’s University, Patchogue, New York, United States

## Abstract

Caregivers increasingly experience disruptions to their social activities due to their caregiving responsibilities. While previous studies have highlighted the negative impact of such disruptions on caregivers’ quality of life, the longitudinal relationship between disrupted activities and the values caregivers place on those activities remains underexplored. Using data from the National Study of Caregiving (NSOC) across three waves (N = 2328), this study examines how disrupted activities and their value change over time and their interaction with caregiver outcomes. Findings from multilevel modeling reveal that caregivers who experienced significant disruptions to their activities while attaching a high value to them reported a rapid increase in negative relationships and burnout over time. Conversely, caregivers who experienced high disruption but placed lower value on these activities showed a more moderate increase in both negative relationships and burnout. Caregivers with lower disruption and lower attachment to activities experienced only a slight increase in these outcomes. These findings underscore the critical role that the interruption of activities and the value placed on those activities play in shaping caregivers’ mental health outcomes. From a practical perspective, this study highlights the importance of considering the meaning caregivers attach to their activities when developing interventions to support their mental well-being. Practitioners should be aware that caregivers who find meaning in their social activities may be at greater risk for burnout and adverse relational outcomes when disrupted. Thus, strategies should focus on providing support for both activity continuity and emotional attachment to activities.

